# Real-World Experience in Treatment of Patients with Non-Small-Cell Lung Cancer with BRAF or cMET Exon 14 Skipping Mutations

**DOI:** 10.3390/ijms241612840

**Published:** 2023-08-16

**Authors:** Urska Janzic, Walid Shalata, Katarzyna Szymczak, Rafał Dziadziuszko, Marko Jakopovic, Giannis Mountzios, Adam Płużański, Antonio Araujo, Andriani Charpidou, Abed Agbarya

**Affiliations:** 1 Department of Medical Oncology, University Clinic Golnik, 4204 Golnik, Slovenia; 2 Medical Faculty Ljubljana, University of Ljubljana, 1000 Ljubljana, Slovenia; 3 The Legacy Heritage Cancer Center & Dr. Larry Norton Institute, Soroka Medical Center, Ben Gurion University, Beer Sheva 84105, Israel; 4 Department of Oncology and Radiotherapy and Early Phase Clinical Trials Centre, Medical University of Gdańsk, 80-210 Gdańsk, Poland; 5 Department for Respiratory Diseases Jordanovac, University Hospital Centre Zagreb, 10 000 Zagreb, Croatia; 6 Clinical Trials Unit, Fourth Oncology Department, Henry Dunant Hospital Center, 115 26 Athens, Greece; 7 Department of Lung Cancer and Chest Tumours, The Maria Skłodowska-Curie National Research Institute of Oncology, 00-001 Warsaw, Poland; 8 Department of Medical Oncology, CHUPorto—University Hospitalar Center of Porto, 4099-001 Porto, Portugal; 9 Oncology Unit, 3rd Department of Medicine, “Sotiria” Hospital for Diseases of the Chest, National and Kapodistrian University of Athens, 106 79 Athens, Greece; 10 Department of Oncology, Bnai-Zion Medical Center, 47 Golomb Avenue, Haifa 31048, Israel

**Keywords:** non-small-cell lung cancer, BRAF V600E mutation, cMET exon 14 skipping mutation, real-world data, targeted therapy, first-line treatment

## Abstract

BRAF and cMET exon 14 skipping are rare mutations of NSCLC. The treatment sequence in these cases for the first and second line is not clear. An international registry was created for patients with advanced NSCLC harboring BRAF or cMET exon 14 skipping mutations, diagnosed from January 2017 to June 2022. Clinicopathological and molecular data and treatment patterns were recorded. Data on 58 patients, from eight centers across five countries, were included in the final analysis. We found that 40 patients had the cMET exon 14 skipping mutation and 18 had the BRAF V600E mutation. In total, 53 and 28 patients received first- and second-line treatments, respectively, among which 52.8% received targeted therapy (TT) in the first line and 53.5% in the second line. The overall response rate (ORR) and disease control rate (DCR) for first-line treatment with TT vs. other treatment such as immune checkpoint inhibitors ± chemotherapy (IO ± CT) were 55.6% vs. 21.7% (*p* = 0.0084) and 66.7% vs. 39.1% (*p* = 0.04), respectively. The type of treatment in first-line TT vs. other affected time to treatment discontinuation (TTD) was 11.6 m vs. 4.6 m (*p*= 0.006). The overall survival for the whole group was 15.4 m and was not statistically affected by the type of treatment (19.2 m vs. 13.5 m; *p* = 0.83).

## 1. Introduction

Lung cancer (LC) is the main cause of mortality worldwide and represents approximately 18% of total deaths from cancer [1,2]. Non-small-cell lung cancer (NSCLC) accounts for about 85% of lung cancer and, despite improvements in early detection, NSCLC is often diagnosed at advanced stages, where patients have a poor prognosis [3,4].

The recent advances in cancer molecular biology have deciphered the oncogenic processes in many types of malignant diseases, including LC. The Lung Cancer Mutation Consortium has elucidated the molecular heterogeneity of NSCLC, discovering molecular alterations in key regulatory pathways that spearhead the malignant process, which, in turn, makes their effective targeting possible and the consequent impairment of cancer growth [5,6]. These molecular alterations are associated with driver mutations. In NSCLC, there are high rates of somatic mutations and genomic rearrangements. Although less frequent, rare mutations are a growing concern. The latest version (3.2022) of the National Comprehensive Cancer Network (NCCN) guidelines for NSCLC recommends that it is important to detect less frequent epidermal growth factor receptor (EGFR) mutations, such as point mutations in exon 18 (G719X), exon 19 insertions, substitution mutations in exon 20 (S768I), exon 20 insertions and substitution mutations in exon 21 (L861Q); human epidermal growth factor 2 (HER2) mutations, such as exon 20 insertions and exon 19 and exon 20 substitutions; Kirsten rat sarcoma virus (KRAS) p.G12C single-nucleotide variations; anaplastic lymphoma kinase (ALK), c-ROS proto-oncogene (ROS1) and rearranged during transfection (RET) rearrangements; B-type Raf kinase (BRAF) V600E mutations; and neurotrophic tyrosine kinase (NTRK) 1/2/3 gene fusion mutations and mesenchymal epithelial transition factor (MET) exon 14 skipping mutations [7,8].

Nowadays, it is possible to identify driver mutations through different methods of molecular analysis. Advanced assays with hybrid capture next-generation sequencing (NGS) enable us to discover a wide range of common and less common mutations, but they are not universally used. Instead, other techniques are routinely applied, such as fluorescence in situ hybridization (FISH), immunohistochemistry (IHC), Sanger sequencing, point-of-care testing or laboratory-developed tests based on PCR technology [9,10]. BRAF and cMET exon 14 skipping mutations are very rare genetic alterations in NSCLC, with incidences of 1–5% and 2–4%, respectively [10].

Genetic assessment in LC varies among different countries; therefore, it is necessary to obtain a comprehensive profiling of genetic mutations of the patients to guide diagnosis and treatment approaches for NSCLC [11]. As a result, many patients could become eligible for targeted therapies (TTs), such as specific monoclonal antibodies or tyrosine kinase inhibitors (TKIs) [12]. Using TTs in the first-line setting has been shown to bring about an improvement in progression-free survival (PFS) and overall survival (OS) in common mutations like EGFR and ALK [13,14].

The treatment paradigm in other less common NSCLC mutations, like MET and BRAF, is less clear due to their rarity and the absence of phase III clinical trials in this subset of the patient population comparing TTs to the standard of care (SOC) [15]. Both mutations affect the downstream signal pathways. MET mutation results in overexpression of its gene product—hepatocyte growth factor receptor (HGFR) [16]. Meanwhile, BRAF activates the MAPK/extracellular-signal-regulated kinase (ERK), leading to cell proliferation, migration, invasion and metastasis [17]. Treatment strategies aim to interfere with these downstream pathways [18,19].

TTs for MET mutations are divided into three types: type I and II are in clinical use, while type III inhibitors are still in preclinical experimental phase [20]. Type I inhibitors are tyrosine kinase inhibitors (TKIs), which bind competitively to the ATP pocket of the receptor, e.g., crizitonib (type Ia), capmatinib and tepotinip (type Ib). Type II inhibitors include cabozantinib, which binds to a hydrophobic pocket adjacent to the ATP binding site [20].

The phase I/II clinical trials GEOMETRY mono-1 [21] and VISION [22] have shown significant clinical activity in patients treated with capmatinib and tepotinib, respectively, including a tolerable toxicity profile. While in the GEOMETRY mono-1 trial, capmatinib was mainly active in treatment-naïve patients, tepotinib showed better antitumor activity in patients treated in advanced lines in the VISION trial [23]. Savolitinib is another MET TKI that has shown efficacy in both first- and second-line settings [24,25]. These MET TKIs have also shown some activity against brain metastasis based on their ability to cross the blood–brain barrier [26].

The use of immunotherapy (IO) as monotherapy or in combination with chemotherapy in first-line treatment in a group of NSCLC MET mutated patients is still controversial despite the high prevalence of positive PD-L1 [27]. This approach is based on small retrospective data collection, which has shown 16–36% response rates, PFS of 1.9–3.4 months and OS of 18 months [28,29,30,31].

Dabrafenib and vemurafenib are a novel generation of BRAF inhibitors [19]. Both drugs have shown antitumor activity in NSCLC patients with the *BRAF* V600 mutation when given as monotherapy [19].

Dabrafenib plus trametinib, a type of MEK inhibitor, when given in combination to NSCLC patients bearing the *BRAF* V600E mutation, has shown an ORR of 64%, PFS of 14.6 months and an OS of 24.6 months [32,33].

Regarding immunotherapy in this group of patients and based on the promising activity with IO plus BRAF targeted therapy in melanoma patients [34], the exact role of IO is still not well defined.

The place of IO treatment in the *BRAF* V600E-mutant NSCLC population is still unclear. Recently, only a few retrospective datasets reporting efficacy, such as longer response after treatment with atezolizumab plus chemotherapy, were published [35,36,37].

Further prospective and real-world data are needed to define the role of the best treatment modality in the first- and second-line settings in the two groups of NSCLC patients described above.

The present real-world trial compared TTs and other systemic treatments in terms of efficacy for the first-line setting in the two groups of mutations (BRAF, MET).

## 2. Results

### 2.1. Clinical and Demographic Characterization of the Cohorts

This data collection identified 133 patients with rare mutations, and further analysis was conducted on a subset of these patients, namely 58 patients who were found to have BRAF and cMET mutations. Of those, 17 (29%) had the BRAF V600E mutation, 1 (2%) had a BRAF translocation and 40 (69%) had the cMET exon 14 skipping mutation (Table 1).

There was no significant difference between the genders. Regarding a history of smoking, almost two-thirds (65%) of the patients had a smoking habit.

The majority of the patients (65%) had background diseases or other chronic conditions. The initial diagnosis stage of the patients included in the study revealed a diverse distribution. Among the participants, 1.7% were diagnosed at stages IB and IIIA, while 3.4% were at stage IIA; stage IIIB accounted for 5.2% of cases and no patients were diagnosed at stage IIIC. Additionally, the majority of the patients (65%) were at stage IVA and 17.2% were diagnosed at stage IVB.

The most common histopathological diagnosis was adenocarcinoma, accounting for 86% of cases. Genetic testing was performed using next-generation sequencing (NGS) in all 58 patients.

PD-L1 levels indicated that 18% of patients had a PD-L1 score below 1%, 34% of patients had a PD-L1 score of 1–49%, and 48% of patients had PD-L1 scores over 50%.

The ECOG performance status of the majority of patients (75%) was 1.

Among the patients with advanced disease, 93% had five or fewer metastatic sites. The most common sites of metastasis included the contralateral lung (50%), bone (45%), pleura (29%), adrenal gland (24%) and brain (21%).

### 2.2. Treatment and Follow-Ups among the Patients

Regarding treatment and follow-up among the patients (Table 2), 9% underwent surgery as part of their treatment, while 10% received stereotactic body radiation therapy (SBRT), and 7% underwent chemoradiotherapy (CRT) as a radical treatment approach. Consolidation with IO was administered to 2% of the patients.

In terms of the first-line treatment regimen, 47% received chemotherapy, while 53% were treated with targeted therapy.

The average number of treatment cycles was 6, ranging from 2.5 to 12 cycles.

Out of 18 patients with BRAF mutation, 1 patient did not receive any systemic therapy. Out of 17 patients treated, 14 patients received targeted therapy (TT) with dabrafenib and trametinib in the first-line setting with an ORR of 78% and DCR of 86%, and only 2 patients had progressive disease as the best response to TT. Meanwhile, 2 out of 3 patients who received either chemotherapy or therapy and progressed then received TT in the second line; the best responses were partial remission and stable disease.

In total, 40 patients were identified with the cMET exon 14 skipping mutation and 36 of them received systemic therapy. First-line treatment with TT was received by 15 patients (4 capmatinib, 10 crizotinib and 1 tepotinib with the following responses: partial response, stable disease and progressive disease in 53%, 13% and 7%, respectively). Meanwhile, 21 patients received either chemotherapy, chemo-IO or IO treatment in the first-line setting and, upon progression, 12 of those patients received second-line TT (2 capmatinib, 8 crizotinib and 2 tepotinib). The best responses in the second line to TT were partial response, stable disease and progressive disease in 58%, 9% and 33%, respectively.

The treatment-related adverse events (TRAEs) following targeted therapy in NSCLC patients were reported to be mostly of grade 1 and grade 2 (*n* = 54, 93.1%). The skin was affected in 43.1% (*n* = 25) of the study participants, followed by fatigue (*n* = 14, 24.1%) and diarrhea (*n* = 11, 18.9%).

There were 42 patients who discontinued first-line treatment; the reasons for discontinuation included death (17%), end of treatment (10%), and disease progression (74%). No patients discontinued treatment due to toxicity.

Progression of the disease was noted at different sites: in the central nervous system, there was disease recurrence in 3% of patients; in only distant sites, in 3% of patients; and in local/regional plus distant sites, in 47% of patients (the same result (47%) was also noted for local/regional progression only).

Among patients progressing after first-line treatment, 27% underwent local radical treatment, and 78% received second-line treatment.

### 2.3. First- and Second-Line Therapy

A statistical analysis was conducted to compare various factors and their associations with different treatment aspects. The number of treatment cycles differed significantly between the groups, with a median of 5.0 cycles (range 2.0–6.5) for the other treatment (mono-chemotherapy, mono-IO, platinum-doublet, chemo-IO) group and a median of 9.5 cycles (range 4.25–14.0) for the targeted therapy group (*p* = 0.01). For the first-line treatment, the median duration was 133.5 days (range 62.3–178.5) for the other treatment group, whereas it was 326 days (range 122–428) for the targeted therapy group (*p* = 0.003) (Table 3). However, there was no significant difference in the duration of second-line treatment between the two groups, with medians of 159 days (range 99.5–285.5) and 194 days (range 106–338) for the other treatment and targeted therapy group, respectively (*p* = 0.51).

Only one patient with the BRAF V600E mutation had initial brain metastases and was treated with stereotactic radiosurgery (SRS) prior to systemic therapy initiation. In total, 11 out of 40 patients with the cMET exon 14 skipping mutation were diagnosed with initial brain metastases, but only 5 of them received local treatment before systemic therapy initiation.

Significant differences were observed for the drug access type of the first-line treatment. Patients receiving other treatments uniformly received first-line therapy through the national reimbursement program, whereas out of the 28 patients receiving targeted therapy, 7% accessed the treatment through extended access programs, 32% through individual reimbursement, 43% through national reimbursement, and 18% through other means like out-of-pocket payment (*p* < 0.001).

### 2.4. Treatments

In the targeted therapy group, 16 patients (57%) died, and 12 patients (43%) were alive at the time of analysis (Table 4). Meanwhile, 15 patients (60%) who received other treatments in the first-line setting experienced death during the study period and 10 patients (40%) were alive at the time of analysis.

### 2.5. Patients’ Survival

The median overall durations of survival for the groups of patients receiving TT (such as biological drugs) or other treatments in the first-line setting were 19.2 months (2.7–35.7 months; 95% confidence interval (CI)) and 13.6 months (8.6–18.5 months; 95% CI), respectively (Table 5).

Overall, considering both groups, the median PFS was 7.9 months (95% CI; ranged from 5.6 to 10.2 months) (Table 5).

## 3. Discussion

The present multicenter real-world study of treatment outcomes in patients with NSCLC harboring rare genetic alterations, i.e., BRAF and cMET exon 14 skipping mutations, has clearly shown the benefit of using targeted therapies, preferably in the first-line setting. Both the response rates and survival are prolonged when targeted agents are used in comparison to other standard-of-care (SOC) therapies for non-oncogene-addicted NSCLC.

A clear advantage to the use of targeted therapy was seen with dabrafenib + trametinib therapy in the current BRAF V600E mutant NSCLC patient cohort. This TT resulted in an ORR and DCR of 78% and 86%, respectively. Most of the patients received this combination of TT in the first-line setting. About half of the patients bearing the cMET exon 14 skipping mutation received TT in first- and half in second-line therapy, and both demonstrated similar outcomes, with promising ORRs of 53% and 58%, and DCRs of 66% and 67%, respectively. Considering the similarities in response rates, the PFS and OS results were reported together and showed a substantial benefit in time to treatment discontinuation, which almost tripled from 133 to 326 days for patients treated with TT.

While the treatment of EGFR- and ALK-mutated NSCLC is well established, the treatment of other rare mutations is still under exploration. Since both types of molecular alterations studied in this retrospective analysis are rare, occurring in about 3–5% (BRAF V 6000E mutation) and 2–4% of NSCLC (MET exon 14 skipping mutation), robust and large-scale data on the characteristic and treatment of patients are missing, as are phase III randomized controlled trials [38,39].

Comparable to the pivotal phase II trial assessing both first- and further-line targeted therapy with BRAF and MEK inhibitors dabrafenib and trametinib, pretreated patients had an ORR of 68%, mPFS of 10.2 months and mOS of 18.2 months, whereas patients receiving the targeted combination in the first-line setting had an ORR of 64%, mPFS of 10.8 months and mOS of 17.3 months. It should be noted, however, that at the time this study was conducted, treatment with ICI was not yet a SOC for patients with NSCLC, which could partially alter the results [40]. As for the MET exon 14 skipping mutation, more tyrosine kinase targeted agents were studied in the phase II trials, such as crizotinib, capmatinib, tepotinib and savolitinib, and those trials displayed positive results, with ORRs spanning from 32% to 68%, mPFS from 6.8 months to 9.7 months and mOS reaching up to 24.6 months [21,41,42,43]. Further trials are also needed to assess whether patients with cMET amplifications and overexpression will derive the same benefit as patients with the exon 14 skipping mutation, since initial signals show that responses are lower and survival is shorter when using MET TKIs [44]. A recent meta-analysis showed a clear benefit of MET TKIs when treating patients diagnosed with advanced NSCLC with the MET exon 14 skipping mutation, resulting in an ORR of 39% and DCR of 78%; when taken together with other MET alterations, the ORR and DCR were 28% and 69%, respectively [18].

Retrospective data have shown a quite consistent benefit of the dabrafenib + trametinib combination treatment for BRAF V600E-positive NSCLC patients, such as our data, with an ORR and DCR of 53% and 85%, respectively, mPFS ranging from 5.0 to 17.5 months and mOS from 10.8 to 22.5 months [33,45,46].

As for patients with the cMET exon 14 skipping mutation, retrospective real-world data show promising results with either crizotinib treatment (mPFS 12.4 months, mOS 22.8 months) or capmatinib therapy (mPFS 9.5 months, mOS 18.2 months) [47,48]. Moreover, a substantial benefit is noted when using the MET inhibitor capmatinib in patients with brain metastases, demonstrating an 85% response rate both systemically and intracranially, even without the primary use of radiotherapy [49]. These data are further confirmed by the meta-analysis, which proved a high intracranial response rate of 95% [18].

New treatment options are underway, mostly phase I/II trials assessing the efficacy of dual or triple inhibition for BRAF, such as encorafenib + binimetinib or vemurafenib + cobimetinib ± atezolizumab [50]. The latter is already showing promising results, where 28 NSCLC BRAF mutant patients have shown an ORR of 18% and a median OS of 13.2 months [51]. The benefit was more prominent in the first-line treatment setting. Clinical trials assessing monoclonal antibodies are underway that target the BRAF mutation [52].

MET inhibitors are also found in further clinical development, both small-molecule TKIs such as merestinib, glesatinib and TPX-0022, as well as antibody-based therapies against MET/HGF, such as Sym-015, telisotuzumab vedotin or combining both MET TKIs with ICI therapy [52,53].

Some retrospective analyses report that patients with oncogene-addicted NSCLC with either a BRAF or MET exon 14 skipping mutation might respond to treatment with IO in monotherapy [54]. Although only about half of BRAF-mutant NSCLC patients carry the specific class I V600E mutation, treatment with IO is not detrimental in this patient population, as it is with chemotherapy [55].

The current study data showed that only 1 patient with the BRAF V600E mutation received IO monotherapy in the first line, with the best response of stable disease, and 4 patients received IO monotherapy in second line after progression on TT, with 3/4 achieving a clinical benefit. As for the MET exon 14 skipping mutation patients, 6/36 received IO monotherapy as the first-line treatment and there was a 67% DCR, whereas only 1 patient received mono-IO treatment after progression on TT, and that patient had a partial response.

Only one retrospective study showed poorer outcomes, which were noted especially in the class I cohort as opposed to the class II and III (non-V600E mutant) cohort, where they did not see a clear benefit of IO in most patients with BRAF alterations. There were ORRs of 9% and 26% for class I and II/III mutations and a higher hazard ratio (HR) for deceased patients who were ever treated with IO [56].

On the other hand, other retrospective analyses, such as the multicenter ImmunoTarget study, showed a 28% and 56% ORR and DCR, respectively, amongst patients with BRAF mutations treated with IO. Additionally, those patients achieved an mPFS of 3.1 months and mOS of 13.6 months [57]. Similarly, another analysis confirmed an ORR ranging from 25% to 33% for patients with class I and II/III BRAF-mutant tumors, and patients experienced significantly longer mOS when treated with IO (from not reported to 21.1 months). The same study also showed that there was a high prevalence of PD-L1 high expressers of 42% in this population [58]. The most recent series showed an ORR and DCR of 30% and 60%, mPFS of 5 months and mOS of 22.5 months for the BRAF V600E group of patients [59]. Another reason for higher IO efficacy in BRAF-mutant NSCLC, as opposed to EGFR or ALK, is the higher tumor mutational burden (TMB) and more frequent smoking status, as almost half of the BRAF-mutant NSCLC patients are found to be smokers [60]. Similarly, this was shown in the present analysis, with 65% of patients either current or former smokers.

In total, 50% of patients with the MET exon 14 skipping mutation had DC (with only 16% having an OR) in the ImmunoTarget study, with an mPFS and mOS of 3.4 and 18.4 months, respectively [57]. Another case series showed prolonged responses when treatment with IO was applied for patients with the MET exon 14 skipping mutation, with 6/13 prolonged responses, which were maintained for 18–49 months [60]. Furthermore, similar series have shown a moderate ORR with IO monotherapy, ranging from 12% to 36%, but with a DCR of up to 72% and mOS from 13.4 to 18.2 months [56,61]. In the post hoc analysis of the GEOMETRY trial, it was shown that patients previously receiving IO were more sensitive to capmatinib treatment versus those that received chemotherapy in prior lines (64% vs. 32%), which led to the conclusion that this is also an IO-sensitive disease [21].

This study’s limitations arose by virtue of conducting a retrospective analysis of data collected at separate medical centers in different countries, which may have varying access to therapies, clinical trials, etc., along with different health policies.

The weak points in targeted therapy for this type of NSCLC arise from the fact that these two mutations (BRAF and cMET) are very rare; therefore, experience is limited (due to the few patients) and insufficient to provide a clear-cut and unambiguous answer yet to whether first-line treatment in these cases should be targeted therapy or chemotherapy. The reports that there is a clinical response to targeted therapy, immunotherapy in patients bearing these mutations give an indication that we should continue offering this type of treatment. In addition, due to the low incidence and hence the difficulty in recruiting participants to clinical trials both for phase I and phase II, treatments of these rare mutations may eventually be recommended based on phase I/II results due to a lack of phase III clinical trials. Additional difficulty in providing a standard of care for the abovementioned patients arises from the fact that each oncologist has individual experience, though it is usually very limited due to the scarcity of these patients [62].

The recent NCCN Clinical Practice Guidelines in Oncology for Non-Small-Cell Lung Cancer (Version 3.2023, 13 April 2023) [62] detail preferred first-line and subsequent therapy following molecular testing results detecting these mutations. In case of positive *BRAF* V600E mutation, the suggestion is to administer dabrafenib in combination with trametinib as the first-line targeted therapy. For patients harboring the *MET*ex 14 skipping mutation, the preferred first-line therapy is capmatinib as a targeted therapy or tepotinib as a selective inhibitor (in cases where the genetic results are delayed, the recommended immediate planned therapy is systemic chemotherapy and immunotherapy). Obstacles in targeted therapy could account for the low response rate and drug-resistance mechanisms, which may lead to progression [63]. The indications for targeted therapy according to the NCCN Guidelines (Version 3.2023, Non-Small-Cell Lung Cancer) are molecular profiling in biopsies and positive detection of driver oncogenes such as the BRAF V600E mutation, METex14 skipping mutation, EGFR exon 19 deletion or exon 21 L858R, EGFR S768I L861Q and/or G719X, ALK rearrangement, ROS1 rearrangement or RET rearrangements [62].

Future prospects for the use of anticancer drugs in the treatment of NSCLC BRAF mutation or cMET exon 14 skipping mutation are under investigation and clinical trials testing the combination of targeted therapy and immunotherapy are needed. Ideally, large trials would provide better statistical interpretation of the results; however, they may take some time to achieve.

Immune checkpoint inhibitor (ICI) therapy combined with MET TKI is an avenue that is being explored, such as capmatinib and anti-PD1 drugs, e.g., nivolumab [52,64].

An ICI combined regimen with chemotherapy might be a further prospective choice for BRAF V600E-mutated NSCLC [52]. A recent review suggests that a promising regimen for NSCLC bearing the BRAF V600E mutation is ICI (e.g., atezolizumab) plus chemotherapy [19].

BRAF inhibitor targeted therapies using dabrafenib in combination are also being explored for the future.

In addition, more potent inhibitors could be developed [52]. The question remains whether the best treatment approach is sequential, or perhaps combining IO treatment with targeted therapies against BRAF or MET mutations would improve outcomes even further.

## 4. Materials and Methods

### 4.1. Study Design

We conducted a real-world retrospective, multicenter, non-interventional observational study, utilizing secondary data collection from medical records/applicable registries of patients with NSCLC harboring a rare targetable genetic alteration, diagnosed and having started treatment up to June 2022. Next-generation sequencing was employed in detecting the BRAF V600E mutation and MET exon 14 skipping mutation. Eight medical centers from five countries (Croatia, Greece, Israel, Slovenia and Poland) took part in this study. Data were reported on patients with the BRAF V600E mutation and MET exon 14 skipping mutation in this analysis. The characterization of patients comprised of gender, age, smoking habits, background disease, stage at diagnosis, ECOG performance status, genetic testing methodology, PD-L1 score and number of metastatic sites. Patient cohorts were devised according to the specific targeted therapy: first-line treatment of BRAF mutation, first-line treatment of cMET exon 14 skipping mutation and second-line treatment of cMET exon 14 skipping mutation.

### 4.2. Study Population

We sampled adults over the age of 18, who were diagnosed with NSCLC harboring a rare targetable genetic alteration (BRAF V600E mutation or MET exon 14 skipping mutation), diagnosed and having started treatment up to June 2022. Participants provided written informed consent for us to collect anonymized data for the study or the ethics committee provided a waiver for the informed consent form. Exclusion criteria were mixed SCLC or neuroendocrine histology. Given the retrospective design of this study, the necessary approvals were secured from the respective Helsinki committees. In Israel, approvals were obtained with IRB code 0023-22-BNZ for the Bnai-Zion Medical Center and approval number 0316 for the Soroka Medical Center. In Greece, permission was granted under reference number 14042/8-6-18. In Poland, the Independent Ethical Committee of the Medical University of Gdansk granted approval with reference number NKEBN/376/2014 for molecular and clinical research involving patients with lung cancer. In Croatia and Slovenia, ethical approval was not required due to the retrospective nature of the study, which did not involve any interventions.

### 4.3. Statistical Analysis

Descriptive statistics in terms of means, medians, percentages, ranges and interquartile ranges (IQRs) were calculated for all the parameters in the study. The normal distribution of the continuous parameters was determined using the Kolmogorov–Smirnov test. As a result of this test, the Mann–Whitney U test was employed to compare groups. For categorical parameters, the Pearson chi-squared and Fisher exact tests were used. Overall survival and progression-free survival were computed using the log rank test. *p* < 0.05 was considered significant. SPSS version 28 was used for all statistical analysis. Data were analyzed as frequencies (*n*) and percentages (%) or medians and ranges. A confidence interval (CI) of 95% and *p*-values were calculated.

## 5. Conclusions

Targeted therapy is the best choice for NSCLC patients bearing rare mutations. TT was shown to prolong both the duration of treatment and overall survival.

## Figures and Tables

**Table 1:** Patients’ demographics and clinical characteristics (*n* = 58).

Characteristic	Frequency (%)
Gender	
Female	28 (48)
Male	30 (52)
Age at diagnosis (years [range])	66.7 [36–91]
Family history of cancer (*n* = 54)	12 (22)
Smoking habit	
Current	17 (29)
Former	21 (36)
Never	20 (35)
Background diseases	38 (65)
Hypertension	26 (45)
Diabetes mellitus	7 (12)
Peripheral vascular disease	3 (5)
Other chronic conditions	17 (29)
COPD ^1^	5 (9)
Hepatic disease	1 (2)
Stage at initial diagnosis	
IB	1 (2)
IIA	2 (3)
IIB	3 (5)
IIIA	1 (2)
IIIB	3 (5)
IVA	38 (66)
IVB	10 (17)
Histological diagnosis: adenocarcinoma	50 (86)
Mutation type	
BRAF V600E point mutation	17 (29)
BRAF translocation	1 (2)
MET exon 14 skipping mutation	40 (69)
Genetic testing method, NGS ^2^	58 (100)
PD-L1 score (*n* = 44)	
<1%	8 (18)
1–49%	15 (34)
≥50%	21 (48)
ECOG performance status (*n* = 57)	
0	5 (9)
1	43 (75)
2	7 (12)
3	2 (4)
Number of metastatic sites at time of advanced disease (*n* = 57)	
≤5	53 (93)
>5	4 (7)
Brain	12 (21)
Contralateral lung	29 (50)
Lymph nodes, extra-thoracic	13 (22)
Pleural	17 (29)
Pericardial	2 (4)
Bone	26 (45)
Adrenal	14 (24)
Liver	12 (21)
Spleen	1 (1)
Peritoneal	1 (1)

Abbreviation: ^1^ COPD, chronic obstructive pulmonary disease; ^2^ NGS, next-generation sequencing.

**Table 2:** Patients’ treatment and follow-up (*n* = 58).

Treatment	Frequency (%)
Surgery	5 (8.6)
SRBT ^1^	6 (10.3)
CRT ^2^ (radical treatment)	4 (6.9)
Consolidation with immunotherapy	1 (1.7)
First-line treatment (*n* = 53)	
Chemotherapy	16 (30)
Chemo-IO ^3^	1 (2)
IO monotherapy	7 (13)
Targeted therapy (TT)	29 (55)
First-line treatment BRAF mutation (*n* = 17)	
Targeted therapy (TT)	14 (82)
Chemotherapy doublet	2 (12)
IO monotherapy	1 (6)
Best response to first-line TT BRAF mutation (*n* = 14)	
Complete response	1 (7)
Partial response	10 (71)
Stable disease	1 (7)
Progressive disease	2 (15)
First-line treatment cMET exon 14 skipping mutation (*n* = 36)	
Targeted therapy	15 (42)
Chemotherapy	14 (39)
Chemo-IO	1 (3)
IO monotherapy	6 (16)
Best response to first-line TT cMET exon 14 skipping mutation (*n* = 15)	
Partial response	8 (53)
Stable disease	2 (13)
Progressive disease	4 (27)
NA	1 (7)
Best response to second-line TT cMET exon 14 skipping mutation (*n* = 12)	
Partial response	7 (58)
Stable disease	1 (9)
Progressive disease	4 (33)
No. of cycles (*n* [range])	6 [2.5–12]
Reason for discontinuation (*n* = 42)	
Death	7 (17)
End of treatment	4 (9)
Progression	31 (74)
Toxicity	0
Type of progression (*n* = 36)	
CNS disease recurrence	1 (3)
Distant only	1 (3)
Local/regional only	17 (47)
Local/regional and distant	17 (47)
Local radical treatment (*n* = 45)	12 (27)
Second-line treatment	28 (48)

Abbreviation: ^1^ SRBT, stereotactic body radiation therapy; ^2^ CRT, chemoradiotherapy; ^3^ Chemo-IO, combined chemotherapy and immunotherapy.

**Table 3:** Patients’ therapy at first and second line.

	Targeted Therapy	Other Therapies	*p*
Median no. of cycles [range]	9.5 [4.25–14.0]	5 [2–6.5]	*p* = 0.01
First-line median duration of treatment (days) [range]	326 [122–428]	133.5 [62.3–178.5]	*p* = 0.003
Second-line median duration of treatment [range] (days)	194 [106–338]	159 [99.5–285.5]	*p* = 0.51
Drug access type at first line, *n* (%)	*n* = 28	*n* = 25	*p* < 0.001
Extended access program	2 (7)	0	
Individual reimbursement	9 (32)	0	
National reimbursement	12 (43)	25 (100)	
Other	5 (18)	0	
Drug access type at second line	*n* = 15	*n* = 13	NS ^1^
Extended access program	4 (26.7)	0	
National reimbursement	9 (60)	12 (92)	
Other	2 (13)	0	
Private insurance	0	1 (8)	

Abbreviation: ^1^ NS, not significant; *n*, number.

**Table 4:** Patients’ treatment regimens.

	Frequency (*n*)	Death Events (*n*)	Censored (*n*)	Percent (%)
Targeted therapy	28	16 (57)	12	43
Other treatment	25	15 (60)	10	40
Overall	53	31	22	42

**Table 5:** Patients’ survival for the treatment regimens (*n* = 53) (months).

Treatment	Median ± SD ^1^	95% Confidence Interval
Survival		
Biological drugs	19.2 ± 8.4	2–35.7
All other	13.6 ± 2.5	8.6–18.5
Overall	15.5 ± 3.5	8.6–22.3
Progression-free survival		
Biological drugs	11.6 ± 3.2	5.4–17.8
All other	4.6 ± 1.3	1.9–7.2
Overall	7.9 ± 1.2	5.6–10.2

Abbreviation: ^1^ SD, standard deviation.

## Data Availability

The data presented in this study are available upon reasonable request from the corresponding author.
